# Random variation in rectal position during radiotherapy for prostate cancer is two to three times greater than that predicted from prostate motion

**DOI:** 10.1259/bjr.20140343

**Published:** 2014-09-16

**Authors:** J Scaife, K Harrison, M Romanchikova, A Parker, M Sutcliffe, S Bond, S Thomas, S Freeman, R Jena, A Bates, N Burnet

**Affiliations:** ^1^ Department of Oncology, Addenbrooke's Hospital, University of Cambridge, Cambridge, UK; ^2^ Department of Physics, University of Cambridge, Cavendish Laboratory, Cambridge, UK; ^3^ Department of Medical Physics, Cambridge University Hospitals NHS Foundation Trust, Cambridge, UK; ^4^ Department of Engineering, University of Cambridge, Cambridge, UK; ^5^ MRC Biostatistics Unit, Institute of Public Health, University of Cambridge, UK; ^6^ Department of Radiology, Cambridge University Hospitals NHS Foundation Trust, Cambridge, UK

## Abstract

**Objective:**

Radiotherapy for prostate cancer does not explicitly take into account daily variation in the position of the rectum. It is important to accurately assess accumulated dose (*D*_A_) to the rectum in order to understand the relationship between dose and toxicity. The primary objective of this work was to quantify systematic (*Σ*) and random (*σ*) variation in the position of the rectum during a course of prostate radiotherapy.

**Methods:**

The rectum was manually outlined on the kilo-voltage planning scan and 37 daily mega-voltage image guidance scans for 10 participants recruited to the VoxTox study. The femoral heads were used to produce a fixed point to which all rectal contours were referenced.

**Results:**

*Σ* [standard deviation (SD) of means] between planning and treatment was 4.2 mm in the anteroposterior (AP) direction and 1.3 mm left–right (LR). *σ* (root mean square of SDs) was 5.2 mm AP and 2.7 mm LR. Superior–inferior variation was less than one slice above and below the planning position.

**Conclusion:**

Our results for *Σ* are in line with published data for prostate motion. *σ*, however, was approximately twice as great as that seen for prostate motion. This suggests that *D*_A_ may differ from planned dose in some patients treated with radiotherapy for prostate cancer.

**Advances in knowledge:**

This work is the first to use daily imaging to quantify *Σ* and *σ* of the rectum in prostate cancer. *σ* was found to be greater than published data, providing strong rationale for further investigation of individual *D*_A_.

Radiotherapy is a clinically effective and cost effective curative treatment for prostate cancer. The major dose-limiting organ at risk is the rectum, located posterior to the prostate. In our centre, inverse-planned intensity-modulated radiotherapy (IMRT) has been delivered to the prostate since 2007.^1^ We use the TomoTherapy® Hi-Art® System (TomoTherapy Inc., Madison, WI), along with other machines. This system delivers image-guided IMRT (IG-IMRT).

For standard treatment, a kilo-voltage (kV) CT scan is acquired for radiotherapy planning. When the patient comes for treatment each day (usually 20–37 treatments), a lower-resolution longitudinally shorter mega-voltage (MV) CT scan is acquired. The patient is then moved, so that the position of the prostate on the MV scan matches its position on the kV scan, and the treatment is delivered.^1^ At present, no allowance is made for the position of the rectum on the MV CT scan. If this were to be different at the time of treatment compared with the time of planning, then the dose delivered to the organ that day (“delivered dose”) might differ from the planned dose.

A variety of studies have demonstrated rectal motion in patients treated with radiotherapy for prostate cancer.^2–9^ These studies have been small and have tended to rely on a limited number of images acquired during treatment. Several studies have confirmed differences between planned and delivered doses to the rectum.^2,6,7,10,11^ These early data support the hypothesis that accumulated dose (*D*_A_) to the rectum over the course of treatment differs from the planned dose in some patients. Development of methods to accurately estimate *D*_A_ is critical for a better understanding of the relationship between dose and effect. This would allow us to advance radiotherapy for the individual patient and is an important radiotherapy research priority.^12–14^

A major impediment to progress is the need for an automated system to track the material elements of the rectum from day to day, both in order to calculate delivered dose in a timely fashion and to do so for a significant number of patients. We carried out the present study in order to understand the location of rectal voxels during a course of prostate radiotherapy, by parameterizing this organ. We tracked the position of the rectum from day to day over the course of treatment and compared the position each day with that at the time of planning. It was important to describe the daily position and to quantify differences within and between patients.

## METHODS AND MATERIALS

This work is from the VoxTox study.^15^ The ultimate aim of VoxTox is to establish *D*_A_ and its relationship with toxicity in 1920 participants treated with IG-IMRT for head and neck cancer, prostate cancer or a central nervous system tumour. Ethical approval was granted on 4 February 2013. From those consented so far, 10 patients with prostate cancer treated with radical radiotherapy to the prostate gland and pelvic lymph nodes were identified. All 10 had been treated using helical tomotherapy with intensity modulation and daily MV image guidance to a dose of 74 Gy in 37 fractions over 7.5 weeks. Patients were asked to have a comfortably full bladder and empty rectum. Treatment was delivered in the supine position using knee and ankle immobilization. kV planning scans and MV image guidance scans were retrieved from the TomoTherapy archive using in-house software, converted into digital imaging and communications in medicine (DICOM) format and imported into the radiotherapy virtual simulation software ProSoma® (Oncology Systems Limited, Shrewsbury, UK).^16^

A clinical oncologist (JS) and radiologist (SF) adapted recent clinical trial protocols for contouring the rectum and femoral heads on kV scans to incorporate features consistently present and identifiable on MV scans.^17,18^ A single clinician (JS) outlined the rectum and femoral heads on the kV and MV scans for the 10 patients; this eliminated interobserver variability. The entire circumference of the rectal wall was contoured on each slice of each MV scan where it was shown. For rectal contouring, the images were viewed at 1.4-times zoom with 83 HU centre and 520 HU width. The most superior slice to contour was defined as the rectosigmoid junction, the last slice before the bowel turns anteriorly and to the left, confirmed using both axial and sagittal planes. The inferior slice to contour was set as the last slice clearly demonstrating both ischial tuberosities.

The entire circumference of each femoral head was contoured on each slice where it could be seen as separate from the femoral neck. For bone contouring, 1.4-times zoom, 300 HU centre and 1500 HU width were selected. The femoral heads were chosen as fiducials, as they could be reliably identified on both kV and MV scans, and the bones were known to be identical in size on all scans for individual patients. They therefore provided a reference that was consistent within patients across scans and could also be used to compare patients. Contouring of rectum and femoral heads following the same protocol was also performed for the kV scans using standard kV windowing.

The rectosigmoid junction was set as slice 1 on kV and MV scans; this allowed matching of the kV and MV scans at the most superior slice level. kV scans had been acquired with 3-mm longitudinal slice thickness and MV scans with 6-mm thickness. The rectum was divided into lower, middle and upper thirds of equal length, and each slice was assigned to one of these thirds. If the total number of slices did not divide exactly by three, then an extra one or two slices were distributed first to the lower third and then to the middle third.

**Figure 1.**
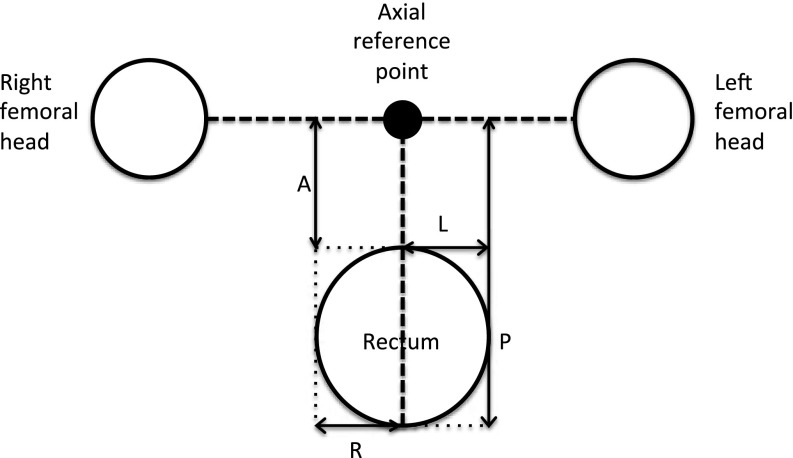
Diagram to illustrate the measurement system from the axial reference point to the anterior (A), posterior (P), right (R) and left (L) extents of the rectum for one slice of one scan in one patient. The centres of the femoral heads were estimated from the centroids of the associated outlines. A point midway between the centres was chosen as the axial reference point, shown in the diagram as a solid black circle. A vertical line was extended from this point towards the rectum, and horizontal lines were extended towards both femoral heads (shown as 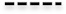). The furthest anterior, posterior, right and left extent of the rectum were identified by enclosing the rectal contour within the smallest possible rectangle (shown as 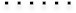). The distance from the axial reference point to the most A, P, R and left L extent of the rectum was measured for each slice of each scan for each patient.

The DICOM data were anonymized, in order to allow their transfer from the hospital network to the Cavendish Laboratory. They were then processed using an in-house application, written in the high-level programming language, Python, as manual measurement of distances on the scans was found to be extremely time consuming. A point midway between the centres of the femoral heads, estimated from the centroids of the associated outlines, was chosen as the axial reference point. The distance from this, to the most anterior, posterior, right and left extent of the rectum, was measured for each slice of each scan for each patient. The axial measurement system is illustrated in Figure 1. The median superior–inferior (SI) position of the femoral head contours was chosen as the SI reference point. The distance from this to the most superior extent of the rectum was measured for each scan for each patient.

Subsequent analysis was performed in Microsoft Excel® (Microsoft, Redmond, WA) and SPSS® (SPSS Inc., Chicago, IL). The MV contours were compared with the kV contours, and methods to parameterize the rectum were developed. These included investigation of variation in rectal position in axial and sagittal directions and axial rectal size. We wanted to assess the merits of incorporating data from all slices *vs* reducing the rectum to thirds. It was important to establish the proportion of rectal voxels that were evaluable by this methodology, in order to plan future imaging protocols. We also wanted to assess variability in contouring using MV scans.

## RESULTS

### Overview

We present detailed results here; a comprehensive summary is given at the end of the Results section in the Summary of key results section.

### Patients and images

#### Scan details and image processing

kV planning CT scans were available for all 10 patients. Complete sets of MV image guidance scans were available for 9/10 patients. In 1 patient, 36/37 scans were available; 1 scan could not be found in the archive (369 scans total). After training, manual contouring of an MV scan took approximately 20 min. Complete contouring for a single patient therefore took 12 h, with 120 h needed for the 10-patient cohort. The Python application produced accurate distances for the contours for 368/369 MV scans. 1 output failed and was omitted; 368 scans were included in the analysis.

#### Number of scan slices showing rectum and extent of rectum visualized

**Figure 2.**
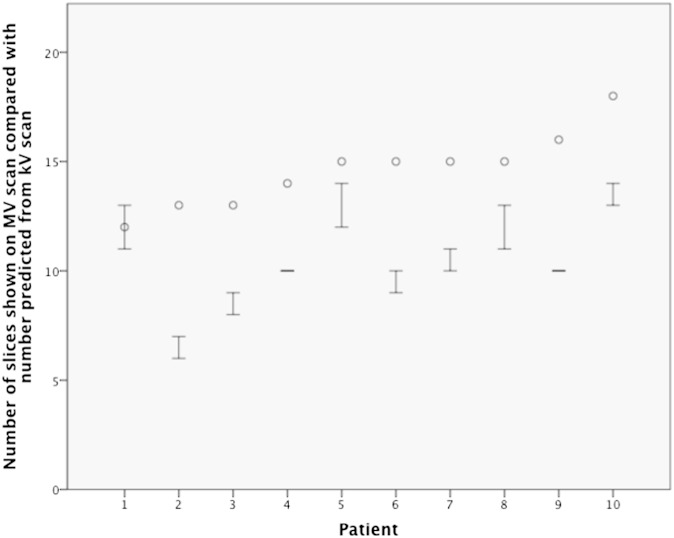
High–low plot of the number of slices showing rectum on the mega-voltage (MV) scans for the 10 patients. The patients are presented in order of ascending number of slices showing rectum on the kilo-voltage (kV) scan. The numbers of slices required to show the entire rectum on the MV scans (*i.e.* half the number of kV slices) are shown as circles. The interquartile ranges, of the actual numbers of slices showing rectum on the MV scans, are shown as bars (25% percentile as the lower bar and 75% percentile as the upper bar).

On the kV scans, the rectum was demonstrated on a median of 30 slices (range, 25–37 slices), representing a median vertical rectal length of 10 cm. This is within the predicted range for this organ.^17^ The rectal lengths that were demonstrated on the MV scans are shown in Figure 2. For each of the 10 patients, at least 1 MV scan contained the projected number of slices required to demonstrate the entire SI extent of the rectum. However, the mean number of slices identified on the MV scans (11 slices) was 4 slices less than the length predicted from the kV scans (15 slices).

The rectosigmoid junction was shown on all MV scans. Hence, each time the actual slice number was lower than that projected from the kV scan, it was slices relating to the lower rectum that were not available. The lower third was missing completely on at least one MV scan for each patient (range, 1–36) and was therefore not able to be assessed by this methodology. Middle and upper thirds were shown on all MV scans, and these parts of the rectum could be analysed in detail. All results that follow relate to scan slices from the middle and upper thirds of the rectum.

### Axial centre of the rectum

#### Overview

The mean position of the centre of the rectum was calculated, in anteroposterior (AP) and left–right (LR) dimensions, for each of middle and upper thirds of the rectum, for each MV scan. These were compared with the corresponding position of these thirds on the kV scan. Differences in position between kV and MV scans were calculated for each day. This allowed estimation of the systematic variation in rectal position between planning and treatment for each patient (mean of the within-patient daily differences) and for the group of 10 patients [standard deviation (SD) of these 10 between-patient means = *Σ*]. It also allowed estimation of the random variation in position for each patient (SD of the within-patient daily differences) and for the group of 10 patients [root mean square (RMS) of these 10 between-patient SDs = *σ*]. Differences between variation in position in the AP and LR directions and between the upper and middle thirds of the rectum were assessed.

#### Position of the axial centre of the rectum

The daily differences in position between kV and MV scans were given as a pair of orthogonal distances: one in the AP direction and one in the LR direction. For the AP direction, distances that were more anterior on the MV than the kV scan were defined as positive and distances that were more posterior on the MV scan were negative. For the LR direction, distances that were more to the left on the MV than the kV scan were defined as positive and distances that were more to the right on the MV scan were negative.

In order to investigate any and all differences in median position between the kV and MV scans, a boxplot was produced for the position of the centre of the rectum for the middle and upper thirds of the rectum, in all patients, in both AP and LR directions, for all treatment days (data not shown). The median position was close to the position on the kV scan at −0.8 mm with an interquartile range (IQR) of −3.4 to 2.0 mm.

#### Variation in position of the axial centre of the rectum

In order to begin to investigate variation in the position between patients, the results from the Position of the axial centre of the rectum section were divided into those for the AP and those for the LR direction and plotted as two histograms. The distributions for both directions were close to normal, with a mean AP position of −2.8 mm [95% confidence interval (CI) −3.3 to −2.3 mm] and a mean LR position of −0.2 mm (95% CI, −0.4 to 0.1 mm). The SD in the AP direction was 6.5 mm and in the LR direction was 3.0 mm.

**Figure 3.**
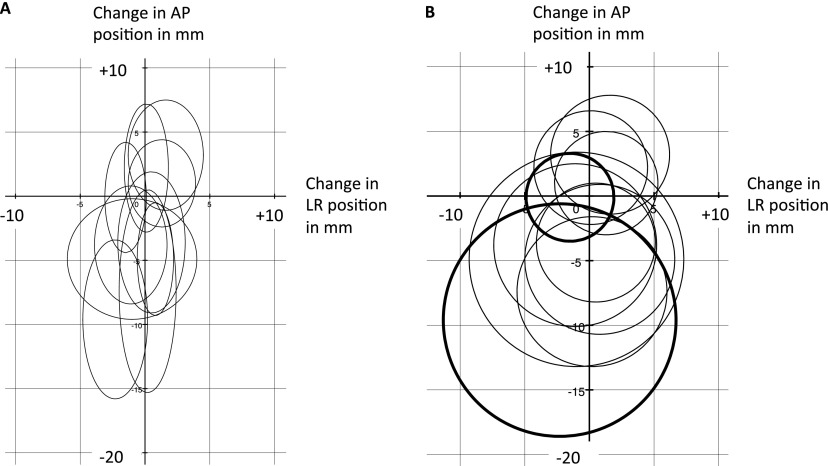
Graphical representation of the mean position of the centre of the rectum and its variation during treatment for the 10 patients, with reference to the position on the kilo-voltage scan (0, 0). The scales for (a, b) are identical. (a) Each centre of an ellipse represents the mean position of the centre of the rectum in a patient, with change in left–right (LR) position shown on the *x*-axis (movement more to the left positive and movement more to the right negative) and change in anteroposterior (AP) position shown on the *y*-axis (movement more anterior positive and movement more posterior negative). The horizontal axes of the ellipses represent two times the standard deviations (SDs) of the LR positions. The vertical axes represent two times the SDs of the AP positions. (b) The data used to produce (b) are identical to those in (a) but have been condensed in order to simplify the diagram and make it easier to visualize differences in systematic and random variation in position of the centre of the rectum between the 10 patients. Each centre of a circle represents the mean position of the centre of the rectum in a patient, with change in LR position shown on the *x*-axis (movement more to the left positive and movement more to the right negative) and change in AP position shown on the *y*-axis (movement more anterior positive and movement more posterior negative). The radii represent a composite interquartile range (IQR) incorporating the results for both the AP and LR directions. The patients with the largest and smallest IQR (3.4 and 9.0 mm) are shown in bold. mm, millimetres.

The mean position was then calculated for each patient in the AP and LR directions in the same way. These mean positions are shown as the centres of the ellipses and circles in Figure 3, and the differences between their locations illustrate systematic variation in this parameter between patients. The mean of the means for the 10 patients was −2.8 mm in the AP direction (95% CI, −5.5 to −0.1 mm) and −0.1 mm in the LR direction (95% CI, −0.9 to 0.7 mm). The SD of the means for the 10 patients (*Σ*) was 4.2 mm in the AP direction and 1.3 mm in the LR direction.

The SD of the AP differences in position between the kV and MV scans was calculated for each patient, along with the SD in the LR direction. The RMS of the 10 AP SDs (*σ*) was 5.2 mm and of the 10 LR SDs was 2.7 mm. In order to visually compare random variation in position between patients, the SD in the AP direction multiplied by two and the SD in the LR direction multiplied by two were used as the axes to construct ellipses as shown in Figure 3a. Owing to overlap and the elliptical shape, these were difficult to understand.

In order to try and simplify the visualization, the data were condensed and a composite IQR was produced, incorporating the results from the Position of the axial centre of the rectum section for both the AP and LR directions. These IQRs were used as the radii to construct circles as shown in Figure 3b (range, 3.4–9.0 mm). They provide an illustrative estimate of the magnitude of random variation in the position of the centre of the rectum within individual patients during the course of treatment.

#### Variation in anteroposterior and left–right directions

In order to assess the differences between the AP and LR directions, ratios were calculated for each patient on the mean AP difference in position (between kV and MV scans) to the mean LR difference in position. For 8/10 patients, the ratio was greater than 1 (median, 4.5; IQR, 2.4–6.9). This gives an estimate as to the magnitude of difference in *Σ* between the AP and LR directions for the middle and upper thirds of the rectum.

In the same way, ratios were calculated for each patient of SD in the AP direction to SD in the LR direction. AP SD was greater than LR SD for 9 of the 10 patients (median, 2.3; IQR, 1.5–2.9). This gives an estimate as to the size of difference in *σ* between the two directions.

#### Variation in position between middle and upper thirds of the rectum

We sought to evaluate whether differences could be detected in *Σ* and *σ* between different thirds of the rectum. The results from the Variation in position of the axial centre of the rectum section were separated into those for the upper and middle thirds. Ratios were calculated for each patient of mean differences in position between the middle and upper thirds. No clear patterns were seen, suggesting that *Σ* does not differ significantly between these thirds.

Ratios were also calculated for each patient of the SDs between thirds. The LR SD for the upper third was greater than that for the middle third in all 10 patients, with a median ratio of 1.8 (IQR, 1.3–1.9). This gives an estimate as to the size of difference in *σ* between the middle and upper thirds of the rectum in this direction. No clear differences were seen for AP SDs between thirds.

### Superior rectum

**Figure 4.**
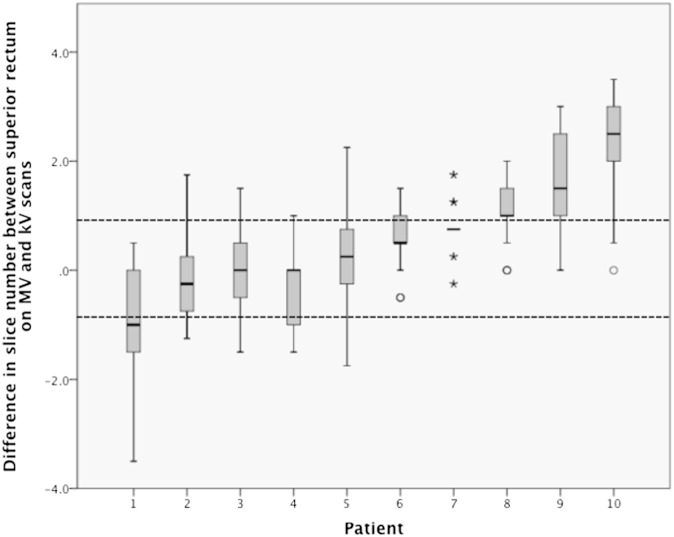
(Supporting material.) Boxplot of the difference in the position of the superior rectal contour between the mega-voltage (MV) and the kilo-voltage (kV) scans for the 10 patients. The patients are presented in order of ascending median difference in position. The dark line represents the median, the bottom of the box the 25th percentile, the top of the box the 75th percentile and the whiskers extend to 1.5 times the height of the box (if this is beyond the range, then extension to maximum/minimum values instead). Outliers are shown as circles and far outliers as asterisks. Dashed lines are drawn at the levels of 1 slice above and 1 slice below the superior rectum on the kV scan; these boundaries contain the interquartile range (IQR) when the 10 patients are combined. Differences can be seen at the individual patient level, with Patients 2, 3 and 5 being the only ones with the IQR contained within one slice above and one slice below the superior rectum on the kV scan.

The SI position of the superior rectum on each MV scan was compared with the position on the kV scan. Positions that were more superior on the MV scan were defined as positive, and distances that were less superior on the MV scan were defined as negative. The median difference in position when all days and patients were combined was 3 mm, with an IQR of −1.5 to 7.5 mm. With an MV slice thickness of 6 mm, this meant that the IQR showed <1 slice variation above and below the superior position on the kV scan. The distribution at the individual patient level is shown in the supporting material in Figure 4.

### Axial size of the rectum

#### Rectal radius

**Figure 5.**
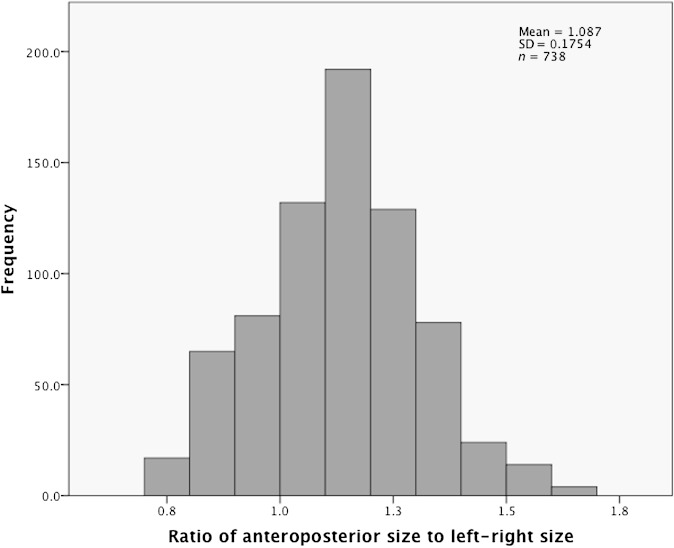
Histogram of the ratio of anteroposterior rectal size to left–right rectal size on the mega-voltage scans for middle and upper thirds in all patients over the 37 days of treatment. SD, standard deviation.

The ratio of AP to LR rectal size on each of the 37 MV scans was calculated for the upper and middle thirds for each patient. A histogram of these results for all patients and treatment days is shown in Figure 5. The mean ratio for this histogram was 1.09 (95% CI, 1.07–1.10) with a SD of 0.18. This meant that, although the rectum could be elliptical in either the AP or LR dimension on a particular slice, provided all slices were taken for a given third, the overall shape was very close to a circle.

This meant that we were able to approximate the rectal circumference to a circle. The rectal radius was calculated as:

$$0.5\times \sqrt{\left(\text{AP}\;\text{size}\times \text{LR}\;\text{size}\right)}$$

**Figure 6.**
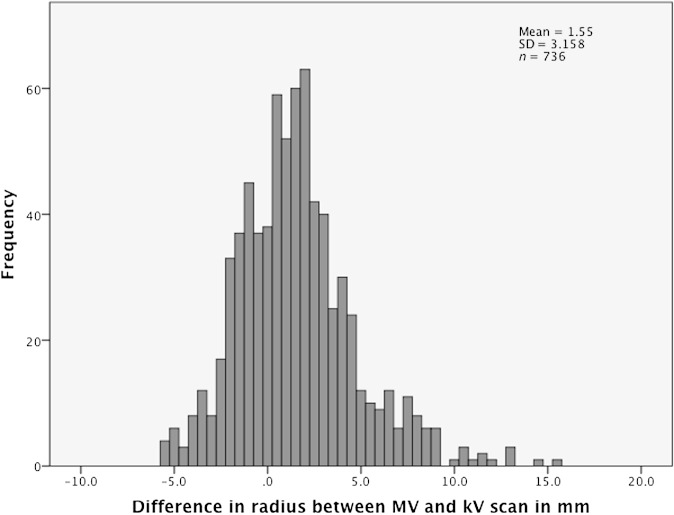
Histogram of the difference between the rectal radius on each of the 37 mega-voltage (MV) scans, and the rectal radius on the kilo-voltage (kV) scan, for middle and upper thirds of the rectum, for the 10 patients. mm, millimetres; SD, standard deviation.

The difference between the rectal radius on each of the 37 MV scans and the rectal radius on the kV scan was calculated for both thirds for each patient. These results are shown as a histogram in Figure 6. The data were skewed owing to a small number of cases where the radius was enlarged by >10 mm between the kV and MV scans (maximum, 15.3 mm). The median radius over the course of treatment, when the 10 patients were combined, was 1.3 mm larger than on the kV scan, with an IQR of −0.6 to 3.1 mm.

A data set was created of the kV scan radius for each of middle and upper thirds for the 10 patients. The median of this data set was 13.3 mm (IQR, 12.3–15.2 mm). This meant that the radius on the MV scan [(13.3 + 1.3)/13.3] was 1.1 times greater, on average, than the radius on the kV scan.

#### Variation in axial size of the rectum

The rectum was initially investigated with results for both upper and middle thirds combined. The median difference in radius between kV and MV scans was calculated for each patient by taking the median of all days and both thirds. The range was −1.4 to 4.2 mm for the 10 patients, reflecting *Σ* in rectal radius between patients. IQR varied between patients (range, 1.8–6.1 mm), corresponding to differences in *σ* between patients.

#### Differences in axial size between thirds

We wanted to establish whether there were differences in axial size between the middle and upper thirds of the rectum. The median MV rectal radius was calculated for each third for each patient; no clear differences were found between thirds.

### Anterior rectal movement

We wanted to assess the rectum at the individual slice level, in order to understand whether reducing the rectum to thirds removed vital information or not. We chose to analyse a single point on the rectal wall, the furthest anterior point, in order to combine information about position and size in one metric.

The change in position of this point was calculated for each slice of each MV scan for the upper and middle thirds of the rectum, for each patient, with reference to the position of that point on the matched slice of the kV scan. Positions that were more anterior on the MV than the kV scan were defined as positive and distances that were more posterior on the MV scan were negative. A histogram of these differences in MV position relative to the kV position was constructed for all slices for all patients. This showed near normal distribution (data not shown). The mean position for this histogram was 0.5 mm more anterior than the position on the kV scan (95% CI, 0.2–0.7 mm). The overall SD was 8.6 mm.

**Figure 7.**
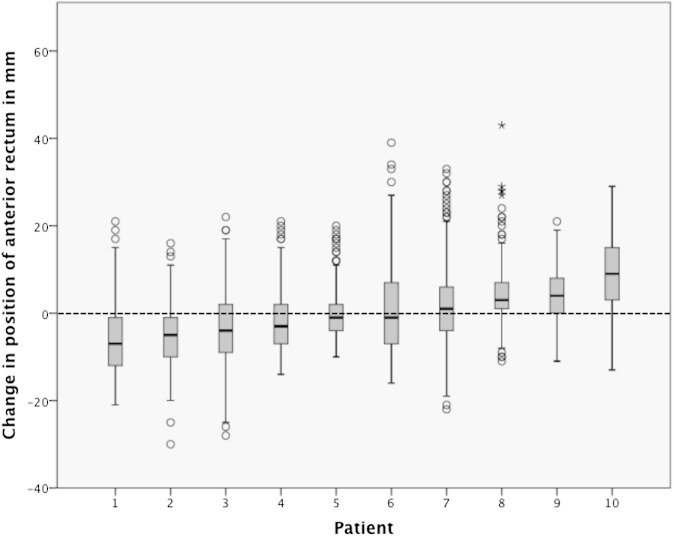
Boxplot of the change in position of the furthest anterior point of the rectum for each slice of each mega-voltage scan (for middle and upper thirds) for each patient, with reference to the position of that point on the matched slice of the kilo-voltage (kV) scan. The patients are presented in the order of ascending median change in position. The dark line represents the median, the bottom of the box the 25th percentile, the top of the box the 75th percentile and the whiskers extend to 1.5 times the height of the box (if this is beyond the range, then extension to maximum/minimum values instead). Outliers are shown as circles and far outliers as asterisks. The dashed line represents the position of the anterior rectum on the kV scan. mm, millimetres.

These data are presented as a boxplot in Figure 7 to illustrate the differences in the position of the anterior rectum, over the course of treatment, within and between patients. The median change in position ranged from 6.5 mm less anterior (Patient 1) to 8.5 mm more anterior (Patient 10). The mean of the means was 0.4 mm more anterior; the SD of the means (*Σ*) was 4.6 mm. The within-patient SDs of these differences in position ranged from 4.7 to 9.3 mm and are illustrated by the whiskers in Figure 7. The RMS of the SDs for the 10 patients (*σ*) was 7.4 mm.

### Variation in contouring

Repeat contouring was performed at a 3-month interval by JS on a random selection of 50 individual slices from these 369 MV scans. Contours were compared using conformity index and mean distance to conformity metrics.^19^ The results for these were 0.83 and 1.0 mm, respectively; these are consistent with data in the literature on contouring variation using kV scans.

### Summary of key results

Manual contouring was time consuming and estimated as 12 h per patient. The MV scans did not reliably show the inferior rectum, and only the middle and upper thirds could be evaluated using this methodology. When the 10 patients were combined, the median position of the axial centre of the rectum was close to its position on the kV scan. Both *Σ* and *σ* of the axial centre were greater in the AP than in the LR direction. *Σ* was found to be 4.2 mm AP and 1.3 mm LR. These results are consistent with previous work in the literature on prostate motion. *σ* was 5.2 mm AP and 2.7 mm LR. These values are higher than those previously found for prostate motion. SI variation was of the order of one slice above and below the position on the kV scan. The rectum could be approximated to a circle in the axial plane, with the median radius during treatment being 1.3 mm larger than on the kV scan. Analysis of the rectum in thirds provided a good approximation of *Σ* at the individual slice level (4.2 and 4.6 mm, respectively). This method appeared to underestimate *σ* with values of 5.2 mm for thirds and 7.4 mm for individual slices. Intraobserver variability of contouring on MV scans was similar to that previously seen for kV scans.

## DISCUSSION

### Extent of rectum visualized

The median vertical height of the rectum on the kV scans was 10 cm, which is within the predicted range for this organ.^17^ This suggests that our contouring protocol, optimized for the longitudinally shorter and lower resolution MV scans, produced contours for the whole rectal height when applied to kV scans. It also suggests that when applied to the MV scans, all slices showing rectum should have been identified. Where the number of contours was lower than that predicted from the kV scan, it was because the whole rectum had not been imaged at the time of the MV scan. We chose patients who were treated with radiotherapy to pelvic lymph nodes as well as the prostate, as our previous work had demonstrated that the image guidance scans for this group were longer in the SI direction than for patients having their prostate alone treated.^20^ The rectum was analysed in thirds, which is the traditional anatomical approach for this organ.^21,22^

For each of the 10 patients, at least one MV scan contained the projected number of slices required to demonstrate the entire SI extent of the rectum. However, the mean number of slices identified on the MV scans (11 slices) was 4 slices less than the length predicted from the kV scans (15 slices). The rectosigmoid was identified on all MV scans for all patients, and therefore where there were slices missing, they related to the inferior rectum. This reflects the fact that the priority for the image guidance scans was to show the position of the prostate and pelvic lymph nodes, which do not extend as far inferiorly as the rectum does.

We were, therefore, able to analyse the position and size of the middle and upper thirds of the rectum, but not the lower third, using these MV scans. We also investigated which parts of the rectum, if they moved, would be most likely to affect *D*_A_. We found that the prostate (high-dose region) was adjacent to the middle third of the rectum for approximately 80% of its length and adjacent to the lower third of the rectum for the remaining 20%. Movement of the middle third is therefore likely to have the greatest effect on *D*_A_. Our initial work suggests that the lower third has less variation than the other two-thirds, as also has been found in other studies.^4,23^ In order to prove this incontrovertibly, for the next cohort of patients recruited to VoxTox, we are extending the inferior border of the MV scans to below the ischial tuberosities, in order to ensure complete coverage of the inferior rectum. This will allow accurate evaluation of this third of the rectum.

### Axial centre of the rectum

#### Position of the axial centre of the rectum

The centre was chosen as the part of the rectum to track from day to day, as it could be reliably and consistently identified on each slice of the MV scans, using the furthest extents in the anterior, posterior, right and left directions. It also permitted examination of movements in the AP and LR directions. Relating the rectum on all scans and all patients to a three-dimensional (3D) bone reference meant that set-up variation was eliminated from this system, allowing more accurate estimates of *Σ* and *σ*.

#### Systematic variation in position of the centre of the rectum

The differences in *Σ* between patients can be estimated from the SD of the individual patient mean positions.^24–26^ The SD for our patient group was 4.2 mm in the AP direction and 1.3 mm in the LR direction.

To our knowledge, two previous studies exist quantifying *Σ* and *σ* in the rectal position during treatment for prostate cancer.^4,23^ Hoogeman et al^4^ performed 8–13 repeat CT scans during treatment for a group of 19 patients who were asked to have a full bladder and empty rectum. The rectum was unfolded and rectal wall displacements were quantified between the planning CT and treatment using co-ordinate maps. They found that AP *Σ* was greater than LR *Σ* for the rectum as a whole, consistent with our results for middle and upper thirds. Stasi et al^23^ looked at 10 patients, who received 2 CT scans per week, prior to their radiotherapy. They were treated supine and were asked to have a full bladder and empty rectum. The difference in position between the anterior, posterior and lateral contours at planning and the average positions of these contours during treatment were compared, with the rectum divided into cranial and caudal halves. The SD for the cranial half was 4.4 mm anterior, 3.0 mm posterior, 3.7 mm right and 2.7 mm left. For the caudal half, the SD was 1.0 mm anterior, 1.9 mm posterior, 1.2 mm right and 1.6 mm left. Our AP *Σ* was similar to the results of Stasi et al for the cranial half and our LR *Σ* was similar to their results for the caudal half. Differences may be owing to their scanning protocol being less frequent than ours.

Most previously published work investigating this area has been based on prostate motion. This has been shown to relate to rectal filling and to a lesser extent, bladder filling and leg motion.^8,9,27–31^ There are 11 studies in the literature that have quantified *Σ* in prostate motion.^3,8,28,30–37^ The median AP variation from these studies was 3.5 mm (IQR, 2.5–4.3) and LR was 1.7 mm (IQR, 0.8–2.0); these values from prostate motion are very similar to our rectal data. Three studies used daily MV scans to assess this motion. Bylund et al^32^ assessed prostate movements on daily cone beam MV CT scans in 24 patients. They found *Σ* of 4.9 mm AP and 2.6 mm LR. Fiorino et al^33^ assessed prostate movements on daily helical tomotherapy MV scans for 21 patients. *Σ* was found to be 3.4 mm AP and 1.6 mm LR. Engels et al^3^ found that a group of 18 patients treated using daily MV image guidance could be split into those with a small and stable rectal cross-sectional area (10 patients) and those with a significantly greater and unstable cross-sectional area. *Σ* of the prostate was 2.4 mm AP in the stable group and 6.1 mm in the unstable group; values for LR variation were not provided. Fiorino et al^37^ investigated patients treated using two different immobilization techniques, either of the pelvis or of the legs. They found that *Σ* was affected by this, with AP variation being 3.5 mm for pelvis immobilization and 2.1 mm for leg immobilization; LR variation was 2.8 and 1.7 mm, respectively.

**Figure 8.**
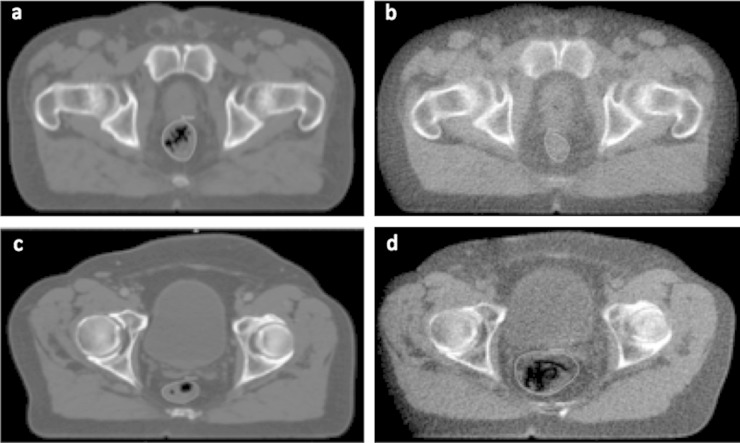
Axial slices at same level from two patients to illustrate differences in mean position at treatment compared with that at planning. (a) Loaded rectum on kilo-voltage (kV) scan from patient with rectal position during treatment of 9.6 mm more posterior than at planning. (b) Empty rectum on Day 1 mega-voltage (MV) scan from same patient as (a). (c) Empty rectum on kV scan from patient with rectal position during treatment of 3.2 mm more anterior than at planning (d). Loaded rectum on Day 35 MV scan from same patient as (c).

The most extreme posterior change in our group was a patient with a mean position of −9.6 mm during treatment. This patient had a loaded rectum at planning (Figure 8a), which had decompressed by the first day of treatment (Figure 8b) and remained emptier during treatment than at planning. The most extreme anterior change was a mean position during treatment 3.2 mm more anterior than the position at planning. Conversely, this patient had an empty rectum at planning (Figure 8c) and was fuller during treatment (Figure 8d). This corroborates work by Hoogeman et al,^4^ who found that mean local AP displacements between planning and treatment ranged from −7.0 to 7.5 mm. In their study, if the planning rectum volume was ≥96 cm^3^, the upper anterior rectal wall shifted posteriorly during treatment, but if the planning volume was <96 cm^3^, the upper rectal wall shifted anteriorly during treatment. Lebesque et al^5^ found that a large rectum at planning was predictive of a large difference between planning and treatment rectal volumes. Rectal distension at planning has also been shown to correlate with an increased risk of biochemical and local relapse of prostate cancer.^38^

#### Random variation in position of the centre of the rectum

An individual patient's SD of daily positional differences provides an estimate of the random variation in position within that patient over a course of treatment (the SD of the random error).^24,26^ The RMS of the SDs of all patients is considered the correct way to determine the group mean of the SD of the random error.^24,26^ This provides an estimate of the differences in *σ* between patients over a course of treatment and for our group was calculated as 5.2 mm in the AP direction and 2.7 mm in the LR direction. The AP SD was greater than the LR SD for 9 out of the 10 patients, with a median AP:LR SD of 2.3. *σ* is most likely to result from differences in rectal filling and emptying from day to day. Hoogeman et al^4^ also found greater AP than LR *σ*.

We found *σ* to be greater than *Σ* in both AP and LR directions. Stasi et al^23^ found the same. The converse was true for Hoogeman et al;^4^ however, this might have been caused by the method of analysis.

There are six studies in the literature that have quantified *σ* in prostate motion during radiotherapy for prostate cancer.^8,30–33,37^ The median AP variation from these studies was 2.7 mm (IQR, 2.4–3.8) and LR was 1.6 mm (IQR, 0.7–2.6). Our results suggest that rectal *σ* is approximately twice prostate *σ* in both AP and LR directions. Fiorino et al^37^ found that *σ* was also affected by immobilization, with AP variation being 3.1 mm for pelvis immobilization and 1.8 mm for leg immobilization, and LR variation being 2.4 and 1.6 mm, respectively.

#### Variation in position between thirds

Greater *σ* was seen in the LR direction for the upper third of the rectum than the middle third, with a median ratio of 1.8 to 1.0. There were no clear differences in *Σ* variation in either direction or AP *σ*. These are in line with the results of Hoogeman et al.^4^

The upper third of the rectum is attached to the rectosigmoid. We propose that the greater LR *σ* for this third is a result of variable filling of the sigmoid colon. Chong et al^22^ assessed rectal movement on weekly cone beam CT (CBCT) scans for 16 patients treated with chemoradiation for rectal cancer. In keeping with our results, they found that *σ* in left and right rectal wall movements was significantly greater for the upper rectum than for the mid rectum.

### Superior rectum

In addition to axial variation, it was important to assess SI variation in rectal position. We were unable to adequately view the inferior rectum on the image guidance scans; this will be tested with a new imaging protocol for the next cohort of participants recruited. Previous work found less SI *Σ* and *σ* for this third than middle and upper thirds.^4^ The median SI position of the femoral head contours was chosen as the SI reference point, meaning that there was a minimum of 3 mm between possible median positions. We found that the most challenging aspect of MV contouring was identifying the superior slice of the rectum, as separate from rectosigmoid, mainly owing to the 6-mm slice thickness. Song et al^39^ looked at differences in kV and MV contours for prostate and seminal vesicles. They also found that the greatest increase in target localization uncertainty between the two scan types was in the SI direction.

These factors meant that our results for SI variation were much less exact than those for axial variation. The IQR was 4.5 mm above and below the kV superior position; this is equivalent to less than one MV slice. It is important to know whether this means that the rectum truly does not significantly vary SI or whether variation has not been adequately captured owing to inaccuracies in this system. Ongoing work is seeking to answer these questions.

### Variation in axial size of the rectum

We found that the median rectal radius was 1.3 mm greater for contours drawn on the MV scans than those on the kV scans, making it 1.1 times as large. Data in the literature regarding this are conflicting. Song et al^39^ found that prostate volumes segmented on MV scans were on average 1.1 times larger than those on kV scans. They attributed this to the lower soft-tissue contrast of the image guidance scans, analogous to larger volumes contoured on CT than on MRI scans.^40,41^ This may not only be a feature of MV CT outlines. The study by Stasi et al^23^ used kV CT scans for contouring, and the average rectal volume during therapy was 8 cm^3^ larger than that at planning. They hypothesized that this may be either owing to reduced compliance with bowel preparation as treatment progressed or to acute proctitis with wall oedema, as also suggested by Stroom et al.^28^

Hoogeman et al,^4^ however, found that the mean rectal volume during treatment was smaller at 74 cm^3^ than at planning (84 cm^3^). Crook et al^36^ found a mean decrease in rectal diameter of 1.5 cm between planning and treatment, and Lebesque et al^5^ found that rectal volume during treatment was 16% smaller than that at planning.^36^ None of these studies used daily imaging. Investigation of time trends in our data is underway to try and clarify the reason for the apparently larger rectum we found during treatment.

### Anterior rectal movement

It was important to examine rectal movement at the individual slice level to see whether this gave additional information about *Σ* and *σ* over and above the mean thirds data. We also wanted to devise a single metric that would allow us to describe both rectal position and size. In order to achieve this, we selected a single point to track in a single direction, namely the most anterior point of the rectum and its AP movements. We chose the anterior rectum for a variety of reasons. Our work showed that the greatest *Σ* and *σ* of the centre of the rectum occurred in the AP direction. Others found that the largest variation in rectal wall movements was at the anterior side.^4^ The anterior rectum is also the part of the rectal circumference closest to the high-dose region; movement of this area is therefore likely to be the most relevant in terms of effects on delivered dose.

*Σ* incorporating anterior movements from all slices from middle and upper thirds was 4.6 mm. *Σ* calculated from AP movements using the thirds data only was 4.2 mm. *σ* for all slices was 7.4 mm and for the thirds was 5.2 mm. These results suggest that analysis of the rectum in thirds was a very good approximation of rectal behaviour at the individual slice level in terms of *Σ*. However, *σ* was greater when calculated using data from all slices. The higher value also represented movement of the anterior rectum, rather than the axial centre. This may indicate that condensing the results into thirds loses some detail about *σ* or may simply reflect the fact that the anterior rectum moves more than the posterior rectum. Hoogeman et al^4^ estimated the greatest *σ* for any part of the anterior side as 8 mm and for the posterior side as 2 mm.

The results suggest that, provided these factors are taken into account, either the axial centre or a point on the rectal circumference could be used to track rectal movement. This information will be important for future work on *D*_A_.

Figure 7 illustrates the differences in *Σ* and *σ* in the position of the anterior rectum between patients. The dosimetric implications of these variations, in terms of dose to the whole rectum, are not yet clear: future work will address this. We propose that *D*_A_ to the anterior rectum for Patient 1 could be lower than the planned dose. In the same way, *D*_A_ to Patient 10 could be higher than that planned.

The voxels within a patient who receives a dose each day depend on the position of the prostate that day; the MV scan is used to move this target into the same 3D position for each treatment fraction as it was in at the time of planning. Variation in rectal position calculated relative to prostate position, rather than our bone reference point, may therefore be less than suggested from our results. Pre-treatment scans were used to assess the rectal position. This does not take into account rectal movements during the actual treatment fraction, which may be significant and may depend on the overall treatment time.^42,43^

### Variation in contouring

A single clinician provided all contours for this study; this eliminated uncertainty owing to interobserver variability in contouring. Estimation of the contouring accuracy of the work is challenging: in this context, other clinicians not used to looking at MV scans would be less likely to provide useful interobserver measurements than would usually be the case with contouring on kV scans.^44^

Previous studies have estimated interobserver conformity index for the rectum on kV planning scans as between 0.77 and 0.81, with the result for kV CBCT being slightly lower at between 0.70 and 0.75.^45,46^ Intraobserver conformity indexes were slightly higher at 0.82 for planning scans and 0.76 for CBCTs.^46^ Our intraobserver conformity index of 0.83 for individual slices was closest to the value previously seen for contouring on kV scans. This suggests that the soft-tissue resolution on the MV scans is certainly good enough to allow for reproducible rectal outlines. Further work is underway to assess whether this level of conformity is also seen for rectal volumes, rather than individual slices, on the MV scans.

### Linking position and dose

Variation in rectal position relative to the prostate is a corollary of delivered dose. We have developed our in-house method for calculating this, based on the MV contours.^47^ Current work is to evaluate *D*_A_ for these 10 patients. We are also developing an automatic method for MV contouring, which is necessary to be able to calculate *D*_A_ for the projected 1200 prostate participants for VoxTox. We are seeking to combine anatomical rule-based finite element modeling with deformable image registration. The future aim, therefore, is to calculate dose differences between patients, rather than positional differences as quantified here.

One small study (38 patients each with an average of 9 scans during treatment) has recently been published investigating the relationship between delivered dose to the rectum and toxicity.^48^ This found that acute toxicity was significantly associated with both rectal volume during treatment and with delivered dose. On average, dose is an excellent biomarker, both of tumour control in the target and toxicity in the normal tissues. This means that, at the population level, dose can be used as a surrogate for toxicity. Performance is not as good at the individual patient level, and more detail is needed. The results from VoxTox are expected to make a major contribution to our understanding of this area, with the potential to modify both toxicity and tumour control on an individual basis in the future.

## CONCLUSIONS

This work shows that the rectum could be tracked from day to day on the MV image guidance CT scans and is the first step towards building a model of this organ for calculation of *D*_A_. *Σ* was similar to the published literature for prostate motion. *σ* of the rectum, however, was found to be approximately two to three times greater than that for the prostate, particularly in the AP direction. These results strengthen the evidence that *D*_A_ may differ from planned dose in some patients treated with radiotherapy for prostate cancer. Development of systems for tracking at the voxel level is underway, in order to understand physical variation in dose between patients. This is essential before biological variation in radiation response can be understood, with the potential for true tailoring of radiotherapy to the individual.

## FUNDING

JS is supported by Cancer Research UK through the Cambridge Cancer Centre. NGB is supported by the NIHR Cambridge Biomedical Research Centre. VoxTox is funded by Cancer Research UK.
